# Retrieval of AH Plus Bioceramic and Ceraseal Versus AH Plus in Endodontic Retreatment

**DOI:** 10.3390/jcm14061826

**Published:** 2025-03-08

**Authors:** Eurok Shim, Jee Woo Son, Jiyoung Kwon, Hyun-Jung Kim, Ji-Hyun Jang, Seok Woo Chang, Soram Oh

**Affiliations:** 1 Department of Dentistry, National Medical Center, Seoul 04564, Republic of Korea; eurokshim@nmc.or.kr; 2 Department of Conservative Dentistry, Graduate School, Kyung Hee University, Seoul 02447, Republic of Korea; julia7348@khu.ac.kr; 3 Department of Conservative Dentistry, Kyung Hee University Dental Hospital, Seoul 02447, Republic of Korea; jyoungkwon@khu.ac.kr (J.K.); kimhyunjung@khu.ac.kr (H.-J.K.); jangjihyun@khu.ac.kr (J.-H.J.); swc2007smc@khu.ac.kr (S.W.C.); 4 Department of Conservative Dentistry, College of Dentistry, Kyung Hee University, Seoul 02447, Republic of Korea

**Keywords:** endodontic retreatment, calcium silicate-based sealer, micro-computed tomography, reciprocating nickel-titanium file, XP-endo finisher, root canal sealer

## Abstract

**Background/Objectives:** Since biomineralization by calcium silicate-based sealers (CSBSs) was reported, retrieving canal filling materials may be challenging during endodontic retreatment due to their adhesion to dentin. This study aimed to evaluate the possibility of removing residual mineral deposits from two kinds of CSBSs compared to the AH Plus Jet (AHJ). **Methods:** Root canals of mandibular premolars were prepared, obturated with the sealer-based obturation method using a WOG medium gutta-percha cone and one of the following sealers: AHJ, AH Plus Bioceramic (AHB), and Ceraseal (CER) (n = 12/group). After 3 weeks, endodontic retreatment was conducted with the WOG files, followed by instrumentation with XP-endo Finisher (XPF). Micro-computed tomography scanning was obtained after canal filling, after retreatment with WOG, and after the use of XPF. The percentage of the removed filling volume was calculated. One-way ANOVA with Tukey’s test and a non-parametric test with Bonferroni’s correction were performed. Root canal dentin after retreatment was examined using a scanning electron microscope (SEM). Results: After supplementary instrumentation with XPF, the mean residual filling volumes for the AHJ, AHB, and CER groups were 1.35 mm^3^, 0.55 mm^3^, and 0.82 mm^3^, respectively. The AHJ group showed greater residual volume compared to the AHB group (*p* < 0.05). The AHB and CER groups demonstrated higher mean percentages of removed filling volume at 94.8%, and 92.5%, respectively, compared to 87.1% for the AHJ group (*p* < 0.05). More mineral deposits were observed in the CER group with SEM. Conclusions: AHB and CER are retrievable during endodontic retreatment, with CER preferable due to greater mineral deposits in dentinal tubules.

## 1. Introduction

Calcium silicate-based cement, such as mineral trioxide aggregate (MTA), has been introduced as a root-end filling material in surgical endodontics due to its excellent sealing ability and biocompatibility [1]. The hydration products of tricalcium silicate and dicalcium silicate powders of MTA are calcium hydroxide and calcium silicate hydrate gels [1,2]. Upon setting, calcium silicate-based cement releases calcium ions, exhibits an alkaline pH, and induces the formation of mineralized tissue [1]. Notably, hydroxyapatite-like crystals are formed when calcium hydroxide reacts with phosphate-containing fluids [3]. Calcium silicate-based sealer (CSBS), derived from calcium silicate-based cement, demonstrates cytocompatibility, alkalinity after the setting reaction, calcium-releasing capacity, and the ability to induce osteogenic marker expression in stem cells [4,5].

The single-cone technique, also referred to as sealer-based obturation method, involves a gutta-percha cone that matches the final preparation size [6]. Along with the use of CSBSs, sealer-based obturation has gained popularity due to its simplicity when compared to lateral compaction or continuous-wave condensation techniques [7,8,9].

The essential characteristics of an ideal root canal sealer comprise dimensional stability, low solubility, thin film thickness, appropriate setting and working times, biocompatibility, antibacterial properties, and retrievability as necessary [10]. Ceraseal (CER; Metabiomed, Cheongju, South Korea), a single syringe-type CSBS, is composed of tricalcium silicate, dicalcium silicate, and tricalcium aluminate and contains zirconium oxide as a radio-pacifying agent, accounting for 45–50% of its composition [11]. CER conforms to the International Organization for Standardization (ISO) standards for flow and radiopacity. Additionally, it exhibits favorable cytocompatibility, calcium release, and alkalizing activity up to 28 days [12,13]. Canal filling with CER, achieved through either the single cone technique, continuous wave of condensation, or carrier-based technique, showed success rates ranging from 83 to 99% [14,15].

The AH Plus Bioceramic (AHB) (Dentsply Sirona, Ballaigues, Switzerland) is another recently introduced CSBS. Unlike many CSBSs containing both di- and tricalcium silicates, AHB exclusively incorporates tricalcium silicate as its reactive component and dimethyl sulfoxide as the vehicle [11]. AHB contains zirconium oxide as a radio-pacifier, constituting 50–70% of its composition [11]. AHB meets the ISO 6876:2012 standard in terms of flow and radiopacity [16]. Its cytocompatibility surpasses that of AH Plus (Dentsply, De Trey, Konstanz, Germany) [5,17]. Samples of AHB immersed in distilled water exhibit alkalinity after 28 days [17]. AHB releases a higher amount of calcium ions than AH Plus but lower than the EndoSequence BC sealer (Brasseler, Savannah, GA, USA) [18].

The failure rate of root canal treatments ranges from 10 to 22% [9,19,20]. The persistence of necrotic tissue or bacteria within the retained filling material of the root canal can lead to treatment failure [21]. The primary objective of endodontic retreatment is to eliminate microorganisms and byproducts responsible for periapical pathosis, ensuring effective disinfection and achieving fluid-tight obturation throughout the root canal system [22]. Consequently, the removal of the previously filled material is crucial for successful endodontic retreatment. An in vitro study revealed that a mineralized deposit was formed on the root canal and was filled using a gutta-percha cone with CER and AHB, followed by immersion in the phosphate-buffered saline for 14 days [23]. Both CER and AHB promoted osteoblastic differentiation of mesenchymal stem cells and exhibited calcium deposition in the matrix [4,24]. Therefore, the removal of filling material during endodontic retreatment appears to be challenging due to the formation of calcific deposits either at the apical region or along the root canal wall. Any residual mineral deposits left after retreatment may hinder penetration of a sealer during the subsequent root canal filling. Therefore, assessing the possibility of removing residual mineral deposits from CSBS is a prerequisite for its use in root canal obturation.

Previous studies have reported that root canals of mandibular premolars filled with CSBSs, such as BioRoot RCS (Septodont, Saint-Maur-des-Fossés, France), TotalFill bioceramic sealer (FKG Dentaire SA, La Chaux-de-Fonds, Switzerland), and Endo C.P.M. sealer (EGEO, Buenos Aires, Argentina), exhibited less residual material after endodontic retreatment compared with AH Plus [25,26,27]. Conversely, Oltra et al. [28] reported that the EndoSequence BC sealer had significantly more residual filling material than the AH Plus sealer. Romeiro et al. [29] reported no substantial difference in the remaining filling material after retreatment between groups filled with EndoSequence BC sealer and AH Plus. These conflicting findings contribute to the ongoing debate regarding the retrievability of CSBS for endodontic retreatment. Furthermore, studies on the retrievability of CER and AHB during endodontic retreatment have been scarcely conducted.

The XP-endo Finisher (XPF) (FKG Dentaire, La Chaux-de-Fonds, Switzerland) is specifically designed to enhance final irrigation and cleaning efficacy [30]. Constructed from a highly flexible martensite-austenite electropolished flex NiTi wire (MaxWire; FKG Dentaire, La Chaux-de-Fonds, Switzerland), it features an ISO #25 tip size with zero taper [31]. At room temperature, XPF exhibits a straight line in the martensite phase, but upon exposure to body temperature, it transforms into a semicircular austenite phase [31]. As it rotates within the root canal, XPF employs a scraping action in the apical 10 mm; therefore, it has been used in endodontic retreatment as a supplementary instrument to remove filling material [31,32,33]. XPF considerably improved the removal of filling material remnants after initial retreatment in an oval-shaped canal [34]. The expansion of the XPF at body temperature as well as its helical movement inside the root canal could reach areas that other instruments might not be able to access without damaging the root anatomy during retreatment [31,34].

This study aimed to compare the retrievability of two types of CSBSs (AHB and CER) during retreatment with an epoxy resin-based sealer (AH Plus), utilizing micro-CT analysis, and to investigate the impact of additional XPF usage on the removal of filling materials. The null hypotheses tested were as follows: (i) there were no differences in the ratio of the removed filling volume after retreatment between different sealers and (ii) there would be no difference in the retrieval efficiency of the sealer after the supplementary use of XPF.

## 2. Materials and Methods

**Figure 1:**
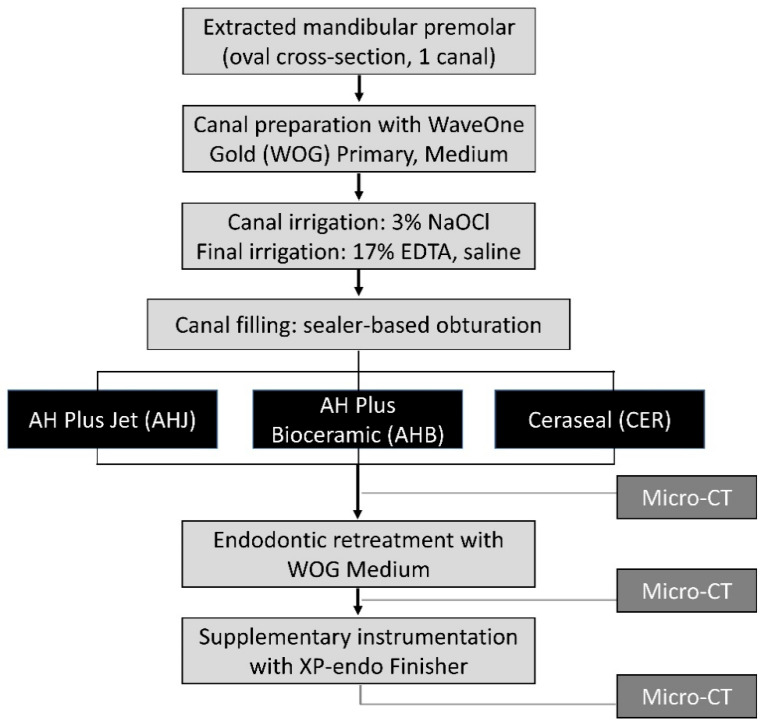
Flowchart of the study.

The entire experimental process is depicted in a flowchart (Figure 1). Sample size calculation was performed based on an effect size of 0.89, derived from a prior study employing a similar methodology [35]. G*Power 3.1.9.7 software (Heinrich Heine, Universität Düsseldorf, Germany) was used; a priori analysis of variance (fixed effects, omnibus, one-way) was selected from the F-test family, with an alpha error of 0.05 and a power (1 − β) of 0.95. The minimum sample size required was eight for each group. In this study, 36 specimens distributed across three groups were designed.

### 2.1. Specimen Preparation and Root Canal Treatment

The study protocol received approval from the institutional review board of Kyung Hee University Dental Hospital (KH-DT23017). This study was performed from August 2023 to December 2023. Permanent human premolars with a single oval canal were selected for inclusion. Teeth exhibiting root caries, severely curved canals, open apices, root resorption, prior endodontic treatment, or cracks were excluded.

All procedures were performed by a single operator, specialized in endodontics, and extensively trained in all the NiTi file systems used. A diamond bur (EX21; Mani Inc., Utsunomiya, Japan) was used to reduce the crown portion, achieving a tooth length of 20 mm with a flat occlusal surface. After the opening of the access, a #10 K-file (Mani Inc., Utsunomiya, Japan) was inserted into the canal until it reached the apical foramen. Root canals were then prepared at the working length using the WaveOne Gold (WOG; Dentsply Sirona, Ballaigues, Switzerland) file at the WOG mode of the X-smart plus engine (Dentsply Sirona, Ballaigues, Switzerland). This involved sequential utilization of the primary (tip size #25, 7% taper for the tip part) and medium (tip size #35, 6% taper for the tip part) files to attain an apical size of #35. Following each instrumentation, apical patency was verified using a #10 K-file, and irrigation was performed using 5 mL of 3% sodium hypochlorite solution. After completion of canal preparation, final irrigation was performed using 1 mL of 17% EDTA, followed by irrigation with 10 mL of saline. Subsequently, the root canals were dried using paper points (Metabiomed, Cheongju, Republic of Korea).

The teeth were randomly divided into three groups, with canals obturated using the sealer-based obturation method with WOG medium gutta-percha cones (Dentsply Sirona), combined with one of the following root canal sealers: AH Plus Jet (AHJ) (Dentsply Sirona; group AHJ), AHB (group AHB), and CER (group CER) (n = 12 each). The composition of the three root canal sealers is listed in Table 1. An AHJ, provided in a self-mixing tip attached to a syringe, was utilized for the AHJ group. AHB and CER were administered in a single-syringe format, injected into the root canals using a syringe tip provided by the manufacturer.

Following canal filling, a heated instrument (Duo-alpha II; B&L Biotech Inc., Ansan, Republic of Korea) was used to remove excess gutta-percha points from the root canal orifice. Then, a condenser (DX condenser; DXM Co., Hwasun-gun, Republic of Korea) was gently applied to the cut gutta-percha with pressure at the orifice level. To ensure the quality of the root canal filling, periapical radiography was performed in both the buccolingual and mesiodistal directions. The coronal area was filled with a temporary filling material (Quicks; DentKist, Gunpo, Republic of Korea). Subsequently, all specimens were stored at 36.5 °C with 95% humidity for three weeks to allow the complete setting of the sealer.

### 2.2. Endodontic Retreatment

Endodontic retreatment was conducted in two phases. Initially, WOG primary and medium files were employed to remove filling materials from the root canals. Retreatment was deemed complete when the WOG medium file reached the working length, with apical patency being verified with a #15 K-file. After each use of NiTi file, root canals were irrigated with a 3% sodium hypochlorite solution.

Following retreatment with the WOG files, additional instrumentation involving the XPF was performed. The XPF was rotated for one minute at 1000 rpm, moving slowly up and down over a length of 7–8 mm, up to 2 mm short of the working length. Root canals were irrigated with 3% sodium hypochlorite solution and warmed to 36 °C during instrumentation to facilitate the phase transformation of the XPF.

### 2.3. Micro-CT Scanning

The teeth were scanned using a SkyScan 1273 micro-CT (Bruker micro-CT, Kontich, Belgium) at 110 kV and 136 μA and 0.3° rotation steps at three time-points: after root canal obturation, after retreatment with the WOG file, and after supplementary instrumentation with XPF. The cross-sectional pixel size and intersection distance were 25 μm. Digital images were generated using the NRecon software (version 1.7.5.9; Bruker micro-CT) to reconstruct the axial sections. The reconstructed files after retreatment were overlapped and aligned with the initial filling file as the reference using the DataViewer software (version 1.5.6.2; Bruker micro-CT). Once aligned, the dataset was exported in an image file format and accessed using the CT Analyzer software (CTAn) (version 1.11.0; Bruker micro-CT).

The CTAn program was utilized to establish a region of interest (ROI) to distinguish the tooth from its surrounding structures. After an appropriate grayscale assignment to distinguish between the filling material and root dentin, this ROI was saved. The volume of the filling materials (gutta-percha and sealer) (mm^3^) was quantified by leveraging a three-dimensional (3D) analysis tool, and the resulting image was saved as a 3D model stereolithography file. An identical protocol was then applied to the scans obtained after retreatment using the same grayscale range for quantifying the volume of the filling materials. The residual filling volumes after retreatment with WOG and supplementary use of XPF (mm^3^) were measured. Initial filling volume and residual filling volumes after retreatment with WOG and after the additional use of XPF were compared among three different sealer groups. It was confirmed that there was no significant difference in the initial filling volume between the three groups via one-way analysis of variance (ANOVA). Since the extracted teeth possess inherent anatomical diversity, the ratio of the removed filling volume was utilized to assess the retrievability of the filling materials.

Once the volume of the filling material was determined, the percentage of the removed filling volume after retreatment was calculated as a volumetric ratio using the following formula:

$$\text{Removed filling volume after retreatment with WOG (\%)}=\;\frac{\mathrm{Initial}\;\mathrm{filling}\;\mathrm{volume}-\mathrm{residual}\;\mathrm{volume}\;\mathrm{after}\;\mathrm{retreatment}\;\mathrm{with}\;\mathrm{WOG}}{\mathrm{Initial}\;\mathrm{filling}\;\mathrm{volume}}\times 100$$

$$\text{Removed filling volume after retreatment with WOG followed by XPF (\%)}=\;\frac{\mathrm{Initial}\;\mathrm{filling}\;\mathrm{volume}-\mathrm{residual}\;\mathrm{volume}\;\mathrm{after}\;\mathrm{supplementary}\;\mathrm{use}\;\mathrm{of}\;\mathrm{XPF}}{\mathrm{Initial}\;\mathrm{filling}\;\mathrm{volume}}\times 100$$

Additionally, the percentage of the removed filling volume by supplementary use of XPF was calculated, considering the residual filling material after the use of WOG as 100% as follows:

$$\text{Removed filling volume by supplementary use of XPF (\%)}=\;\frac{\mathrm{Residual}\;\mathrm{volume}\;\mathrm{after}\;\mathrm{retreatment}\;\mathrm{with}\;\mathrm{WOG}-\mathrm{residual}\;\mathrm{volume}\;\mathrm{after}\;\mathrm{supplementary}\;\mathrm{use}\;\mathrm{of}\;\mathrm{XPF}}{\mathrm{Residual}\;\mathrm{volume}\;\mathrm{after}\;\mathrm{retreatment}\;\mathrm{with}\;\mathrm{WOG}}\times 100$$

The percentages of the removed filling volume after retreatment with WOG and the removed filling volume after retreatment with WOG followed by XPF were assessed for both the total root canal length and specific root regions, i.e., the middle-cervical and apical (4 mm of length) regions.

The CTVol software (version 13.2.3; Bruker Micro-CT) was employed to generate representative 3D images after canal filling, retreatment with WOG, and supplementary use of XPF.

### 2.4. Scanning Electron Microscopy Examination

After supplementary instrumentation with XPF, one specimen from each sealer group with minimal residual filling material was selected, and the root canal wall dentin was examined using field-emission scanning electron microscopy (FE-SEM) (Apero S; Bruker, Billerica, MA, USA) at magnifications of ×1500 and ×3000. The specimens were initially fixed with a 2.5% glutaraldehyde solution (Sigma-Aldrich, St. Louis, MO, USA) and then dehydrated. A uniform platinum ion-coated layer was formed on the sample surface using Q150TS (Quorum Technologies, East Sussex, UK). The elemental composition of the particles in the dentinal tubule was analyzed using energy-dispersive X-ray spectroscopy (EDS) (Xflash 6160; Bruker, Kontich, Belgium) at ×3000 magnification.

### 2.5. Statistical Analysis

The initial filling volume, residual filling volumes after retreatment with WOG, and after the additional use of XPF were compared among the three different sealer groups (AHJ, AHB, CER) using one-way ANOVA and Tukey’s test. Normal distribution and homogeneity of variances of the data were verified according to the Shapiro–Wilk test and Levene’s test, respectively. The analysis was performed for the apical region, middle-cervical region, and total canal length, respectively.

The percentages of the removed filling volume after retreatment with WOG (%) were compared among the three different sealer groups by the Kruskal–Wallis test with Bonferroni corrections because the normal distribution of the data was not verified. The analysis was performed for the apical region, middle-cervical region, and total canal length, respectively.

The percentages of the removed filling volume after retreatment with WOG followed by XPF (%) were compared among the three different sealer groups using one-way ANOVA and Tukey’s test. Normal distribution and homogeneity of variances of the data were verified according to the Shapiro–Wilk test and Levene’s test, respectively. The analysis was performed for apical region, middle-cervical region, and total canal length, respectively.

The percentages of the removed filling volume by supplementary use of XPF (%) were compared among the three different sealer groups using Kruskal–Wallis test with Bonferroni corrections. The analysis was performed for apical region, middle-cervical region, and total canal length, respectively.

Finally, three-way ANOVA was conducted to figure out the influence of the sealer (AHJ, AHB, CER), root region (apical, middle-cervical), instrument (only WOG, WOG followed by XPF), and their interactions on the percentage of the removed filling volume.

All statistical analyses were performed at a significant level of 5% using SPSS software (version 29.0; SPSS Inc., Chicago, IL, USA).

## 3. Results

### 3.1. Root Canal Filling Volumes

**Figure 2:**
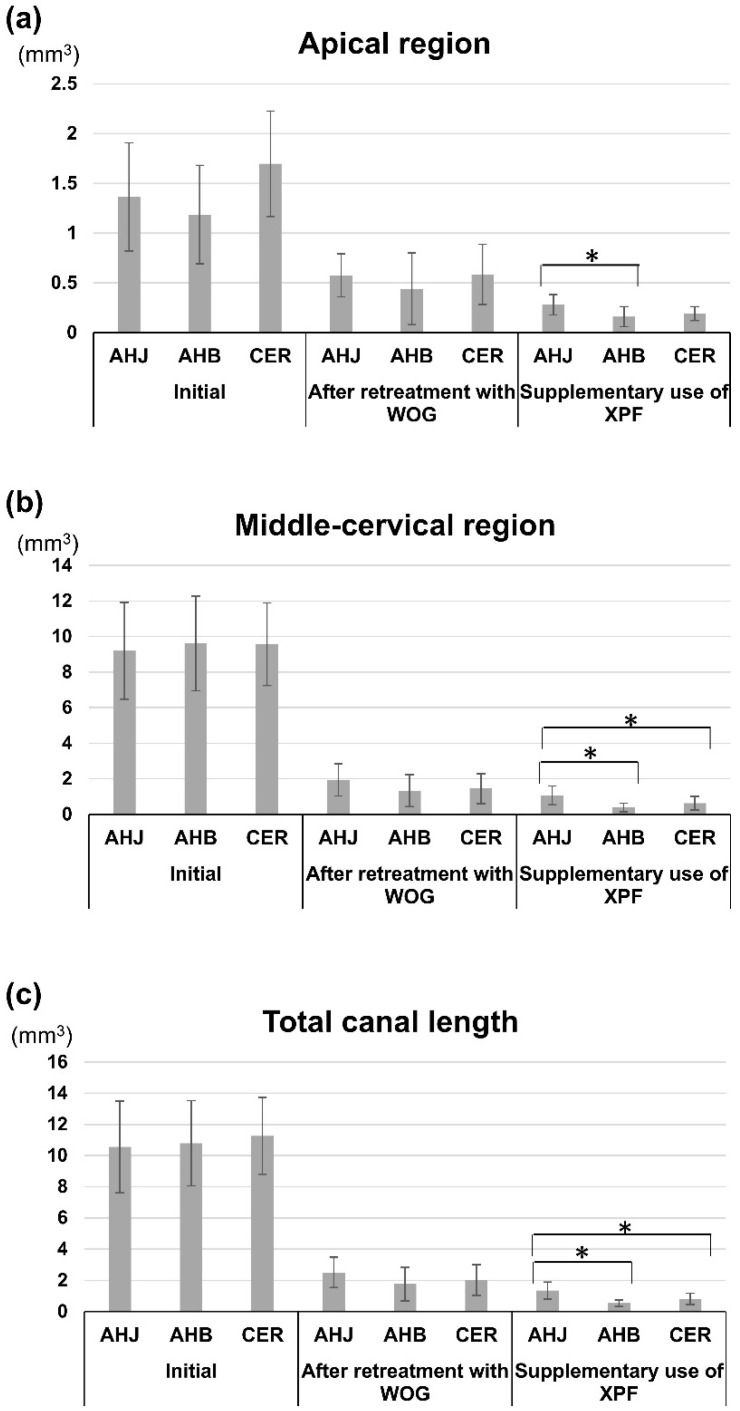
Initial filling volume (Initial), residual filling volumes after retreatment with WOG, and after supplementary use of XPF, respectively (mm^3^), measured in (**a**) apical, (**b**) middle-cervical regions, and (**c**) total canal length. An asterisk indicates a significant difference between groups (*p* < 0.05).

The mean initial filling volumes were 10.56 ± 2.95 mm^3^, 10.79 ± 2.73 mm^3^, and 11.26 ± 2.47 mm^3^ in the AHJ, AHB, and CER groups, respectively. No significant differences were observed in the initial filling volumes among the three groups, either in the apical or middle-cervical regions or in the total length of the canal (*p* > 0.05) (Figure 2a–c) (Table 2).

There was no significant difference in the residual filling volume after retreatment with WOG among the three different sealer groups in terms of the total length of the canal, middle-cervical, and apical regions (*p* > 0.05) (Table 2). After supplementary instrumentation with XPF, the residual filling volume of the AHJ group surpassed those of the AHB and CER groups in the middle-cervical region and in the total length of canal (*p* < 0.05) (Table 2) (Figure 2a–c). The AHJ group had greater residual filling material volume than the AHB group in the apical region after XPF use (*p* < 0.05) (Table 2) (Figure 2b).

### 3.2. Assessment of the Ratio of the Removed Filling Volume After Endodontic Retreatment

**Figure 3:**
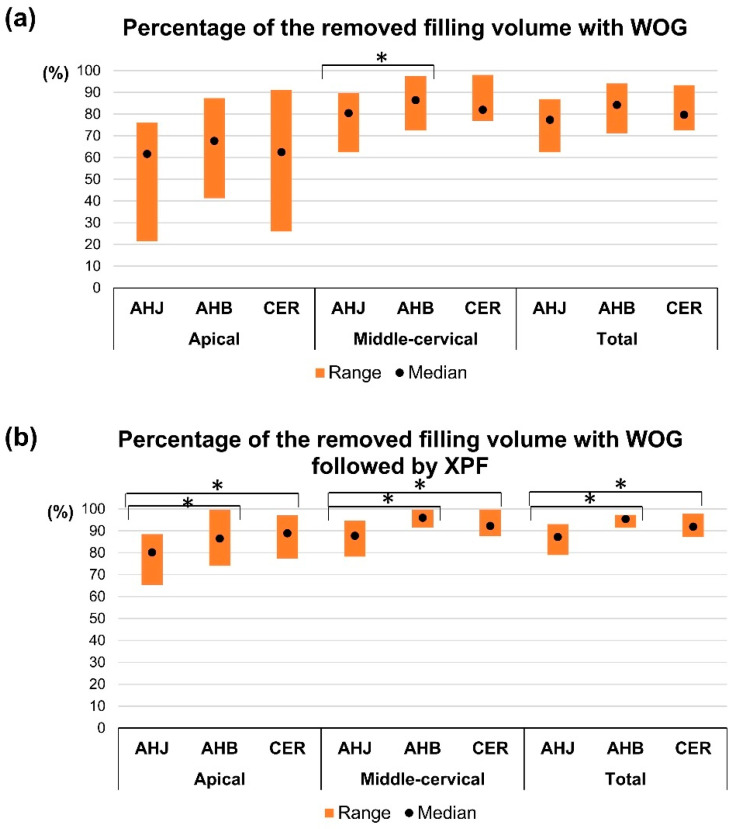
Percentages of the removed filling volume (%) after retreatment with WOG (**a**) and after retreatment with WOG followed by XPF (**b**), considering initial filling volume to be 100%. An asterisk indicates significant difference between the two groups (*p* < 0.05).

The percentages of the removed filling volumes after retreatment with WOG files for the three groups are 76 ± 7.1, 84.4 ± 7.2, and 81.6 ± 7.7% for AHJ, AHB, and CER groups, respectively. The percentage of the removed filling volume of AHB exceeded that for AHJ for the middle-cervical region (*p* < 0.05) (Table 3) (Figure 3a). There was no significant difference in the percentage of the removed filling volume with respect to either the total canal length or the apical region between the groups (*p* > 0.05) (Table 3) (Figure 3a).

The percentage of the removed filling volume after retreatment with WOG followed by XPF for the three groups are 87.1 ± 4.4, 94.8 ± 2.1, and 92.5 ± 3.1% for the AHJ, AHB, and CER groups, respectively. The AHB and CER groups demonstrated greater percentages of the removed volume (%) than the AHJ group for the apical, middle-cervical regions, and total length of the canal (*p* < 0.05) (Table 3) (Figure 3b).

**Figure 4:**
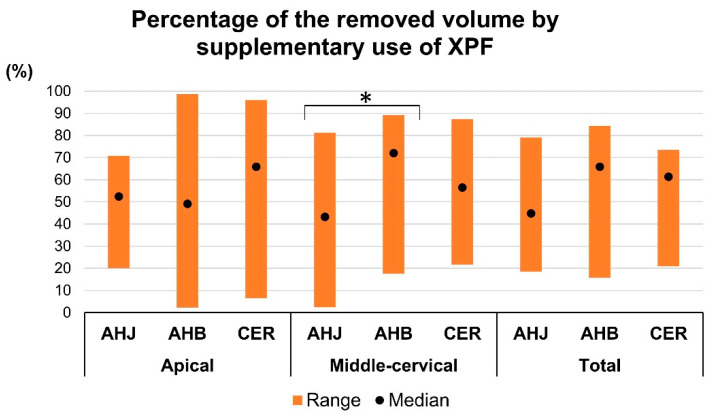
Percentage of the removed filling volume by supplementary use of XPF, considering the remaining filling material after retreatment with WOG to be 100%. An asterisk indicates significant difference between the two groups (*p* < 0.05).

The percentage of the removed filling volume by supplementary use of XPF (%) was calculated, considering the remaining filling volume after retreatment with WOG to be 100%. A greater percentage of filling material was removed by XPF in the AHB group compared to the AHJ group in the middle-cervical region (*p* < 0.05) (Figure 4). There were no significant differences in the apical region and total length of canal among the three groups (*p* > 0.05) (Figure 4).

### 3.3. Three-Way ANOVA Result

There were significant differences in the percentage of the removed filling volume according to sealer, root region, and instrument (Table 4). The interactions between factors were not significant except for ‘root region × instrument’.

### 3.4. Three-Dimensional Visualization of the Roots

**Figure 5:**
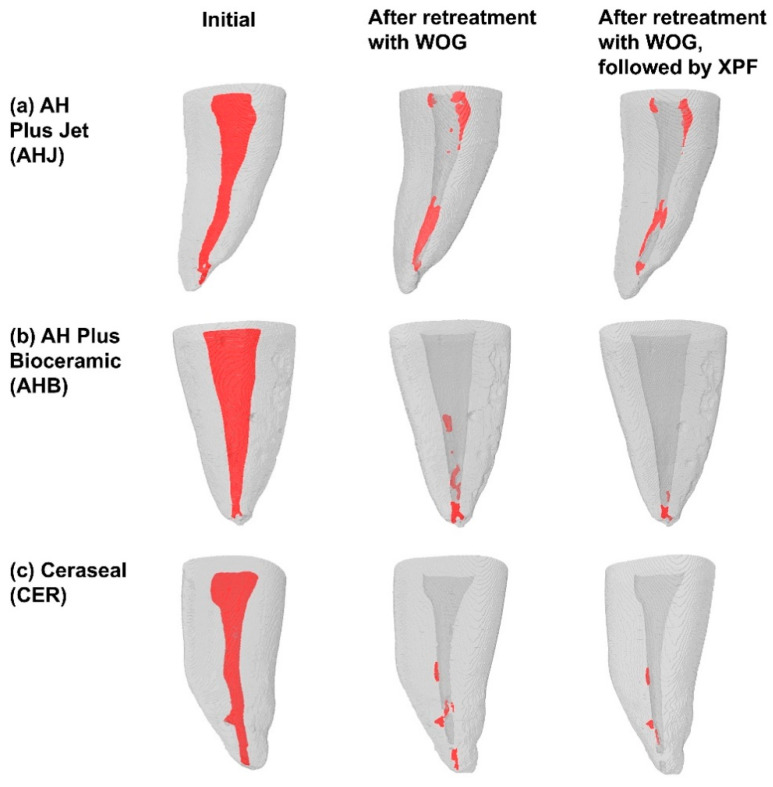
Representative 3D visualization images based on micro-CT scanning before retreatment (initial), after retreatment with WOG files, and after retreatment with WOG followed by XPF in three different sealer groups. (**a**) AHJ; (**b**) AHB; and (**c**) CER. Root canal filling materials were shown in red.

A comprehensive 3D visualization analysis provided evidence of reduced filling material due to the application of WOG and XPF (Figure 5a–c). Furthermore, when comparing different sealers, it was apparent that the AHJ group retained the greatest amount of residue after retreatment (Figure 5a).

### 3.5. FE-SEM Examination

**Figure 6:**
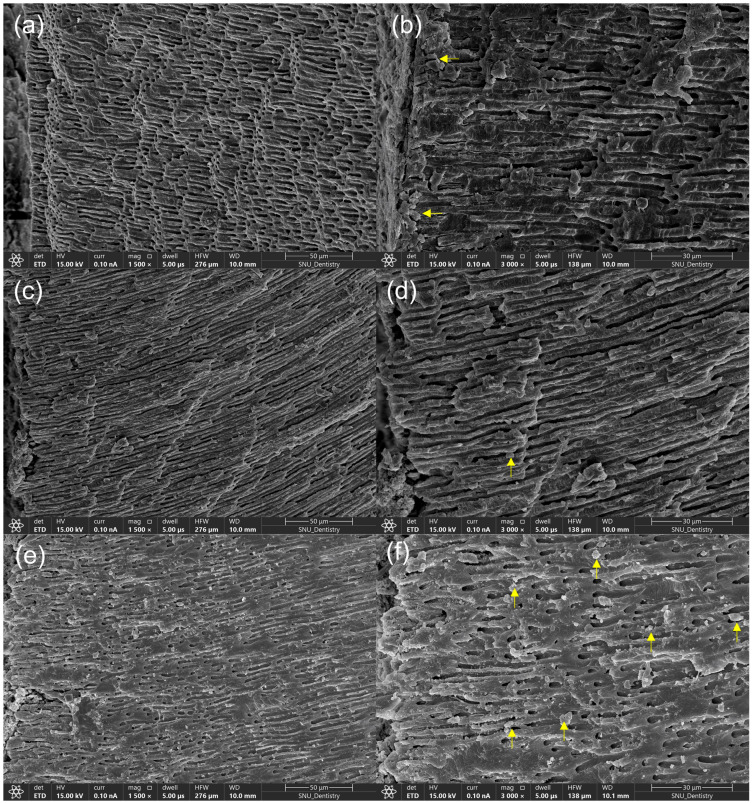
FE- SEM images of the root canal dentin of representative specimen after supplementary instrumentation with XPF in three groups. (**a**,**b**) AHJ group, AH Plus sealer plugged the orifice of the dentinal tubule (arrows); (**c**,**d**) AHB group, a few globular particles were observed within the dentinal tubules (arrows); and (**e**,**f**) CER group, many crystalline structures were observed within the dentinal tubules (arrows). (Magnifications: (**a**,**c**,**e**), ×1500; (**b**,**d**,**f**), ×3000).

Following endodontic retreatment with WOG and the supplementary use of XPF, specimens from the AHJ group showed that the AH Plus sealer plugged the orifice of the dentinal tubules (Figure 6a,b). A few globular particles were observed within the dentinal tubules of mice in the AHB group (Figure 6c,d). Specimens from the CER group exhibited numerous mineral deposits within the dentinal tubules (Figure 6e,f).

EDS was used to analyze the elemental composition and ratio in a specific dentin area, including sealer particles or mineral deposits. The EDS analysis revealed that particles from the AHJ and CER groups contained nitrogen and fluoride, while those from the AHB group did not (Table 5). The AHB and CER groups exhibited higher atomic ratios of calcium to phosphorus, whereas the AHJ group showed a higher ratio of phosphorus to calcium.

## 4. Discussion

Although recently introduced CSBSs are recognized for their biocompatibility, antibacterial effects, and biomineralization ability [12,13,17,18,23], difficulties have arisen in removing these sealers during endodontic retreatment. This study showed that the AHB and CER groups presented greater retrievability compared to the AHJ group. Furthermore, the supplemental use of XPF enhanced removal of the previous filling material. Therefore, the null hypotheses tested were rejected.

In the present study, groups filled with CSBSs, specifically AHB and CER, showed a greater retrievability compared with the AHJ group after retreatment by using WOG supplemented with XPF. This result agreed with previous studies which showed less remaining filling material after endodontic retreatment in the root canals filled with CSBS compared with AH Plus [25,26,27].

The reason for the increased retention of the filling material following endodontic retreatment in the group previously obturated with AH Plus is as follows. Firstly, the presence of a covalent bond between AH Plus and root dentin contributed to the higher amount of remaining material. AH Plus, being an epoxy resin-based sealer, chemically interacts with the collagen network of root dentin by forming covalent bonds between the epoxy rings and exposed amine groups within the collagen network [36].

Secondly, the superior bond strength between AH Plus and root canal dentin may account for the greater residual filling volume. Shieh et al. [37] found that AH Plus exhibited higher push-out bond strength compared to AHB and EndoSequence BC sealer when using a final irrigation protocol of 15% EDTA followed by 4% sodium hypochlorite in single-rooted teeth.

Thirdly, the higher solubility of AHB might facilitate its removal during retreatment. AHB exceeded the limit of the ISO standard even though the sealer disk was allowed to set completely before immersion in water [12,18]. The AHB group had the highest percentage of the removed filling volume after retreatment with WOG (Table 3, Figure 3a). Lastly, the superior wettability of CSBSs allowed the root canal irrigation solutions to deeply infiltrate the sealer mass, reaching the root canal dentin side during the retreatment process. According to Kharouf et al. [38], the water contact angles of AHB and AH Plus were 0° and 64.9°, respectively. The water contact angle of Ceraseal was reported to be 16.8° [39]. In this study, canal irrigation was performed using 3% sodium hypochlorite followed by a final irrigation protocol involving flushing with 17% EDTA and saline. Irrigation with EDTA enhances the removal of the smear layer, thereby improving the penetration of AH Plus into the peritubular dentin [40]. This process increases resistance to bacterial leakage [41] and improves bond strength [42].

Endodontic retreatment of oval-shaped canals is challenging because their noncircular anatomy presents increased complexities and difficult-to-reach areas [43]. In the present study, a reciprocating NiTi file (WOG file) was used to for retreatment. Several previous studies used reciprocating NiTi files in endodontic retreatment and reported that the reciprocating files were unable to completely remove old filling materials [31,44,45,46,47]. Therefore, the effectiveness of supplementary cleaning with XPF was investigated. Retrieval of the filling material was improved in the recess area as well as main canal wall, as illustrated in Figure 5. Our findings are consistent with previous studies that showed the additional use of XPF enhances the removal of old filling materials in the retreatment of oval canals with minimal curvature [34,48] or curved canals [31,32,33]. The XPF, manufactured with MaxWire, offers increased flexibility [30]. When rotating within a canal, the file adapts and conforms to the individual canal through contraction and expansion [48]. As a supplementary method for removing canal fillings, the XPF induced minimal volume change in curved mandibular molars and can be preferred for endodontic retreatment [33].

According to the three-way ANOVA analysis, sealer, root region, and instrument significantly affected the ratio of the removed filling volume. The interaction between root region and instrument also significantly affected the ratio of the filling volume. Removing old filling material in the apical region of the root canal presents challenges when using a NiTi file that matches the canal’s preparation size [31]. The effectiveness of using XPF has been particularly demonstrated in the apical region, as shown in Figure 3 and Figure 5. It may be advisable to enlarge the apical area by using an instrument with a larger tip diameter to enable XPF to perform at its maximum capacity.

CSBS forms intra-tubular tags along with an interfacial mineral interaction layer referred to as the “mineral infiltration zone” [49]. Intra-tubular penetration of CSBSs has been demonstrated in in vitro studies [23,50,51]. Consequently, it can be inferred that the interaction between the calcium silicate-containing sealer and dentin surface might interfere with the complete removal of sealer remnants from the root dentin walls during retreatment [27]. However, in the present study, the AHB and CER groups showed a notably higher percentage of the removed filling volume than the AHJ group after retreatment (*p* < 0.05) (Table 3, Figure 3b). Final irrigation with EDTA before canal filling might cause weaker reaction between root canal dentin and CSBS [25]. In this study, endodontic retreatment was performed three weeks after canal filling, which would be insufficient to form a mineralized layer between the root canal dentin and the CSBS. Among two kinds of CSBSs tested in this study, AHB remained the least filling material after retreatment (Figure 2, Table 2). AHB contains only 5–15% tricalcium silicate, in contrast to the conventional CSBS, which typically contains 7–15% dicalcium silicate and 20–35% tricalcium silicate [11]. Therefore, chemical interaction between AHB and root canal may not be strong enough to resist retrieval during retreatment.

In this study, a premixed CSBS was used in a sealer-based obturation technique with gutta-percha cones which matched the size of the last NiTi file used. Although the instructions of AH Plus recommend lateral compaction or the continuous-wave condensation method, a sealer-based obturation method was employed in this study to minimize the differences originating from the obturation method and the amount of gutta-percha. In addition, an auto-mixed syringe of the AHJ was used to prevent differences in paste content caused by hand mixing.

The primary objective of endodontic retreatment is to eradicate microorganisms and their byproducts that lead to periapical pathosis by removing filling remnants, which obstruct access to microbes in challenging areas. This ensures effective irrigation and medication delivery throughout the entire root canal system [28,29]. Based on the results of this study, it is anticipated that root canals filled with AHJ may require more mechanical and chemical means during endodontic retreatment, thus requiring additional time compared to those filled with AHB.

FE-SEM serves as a valuable tool for examining the surface morphology and texture of materials. In contrast to AHJ, specimens in the AHB and CER groups revealed mineral deposits within the dentinal tubules. Both AHB and CER contain tricalcium silicate in their composition, but CER also contains dicalcium silicate. This difference accounts for the significantly higher number of mineral deposits observed in the CER specimen compared to the AHB specimen. EDS analysis detected elements in the dentin area, including sealer particles, calcium, oxide, and phosphorus, in all specimens. The calcium in the AHJ group specimen could have originated from calcium tungstate in the AHJ. The nitrogen detected in the AHJ specimen originated from a component of AHJ, while in the CER specimen, it was possibly present in the CER thickening agent. Both AHB and CER are CSBSs that contain zirconium oxide as a radiopacifying agent; however, the EDS results did not show zirconium and silica. After the setting reaction of the sealer, the zirconium likely remained on the canal wall, was unable to penetrate the dentinal tubules, and was possibly removed during retreatment.

The strength of this study is the application of micro-CT to assess the removed filling volume through endodontic retreatment. Micro-CT is non-destructive and can be repeated on the same sample. The quantification of the removed filling volume was obtained by overlapping the reconstructed files of initial scan with those of the post-retreatment scan, providing reliable and precise measurements.

CSBS contains calcium silicates that generate calcium hydroxide through a setting reaction [11]. The alkaline pH resulting from CSBS is advantageous because calcium hydroxide promotes bioactivity by facilitating the formation of hydroxyapatite-like materials on the sealer surface [17,18]. The limitation of this study is that endodontic retreatment was performed three weeks after canal filling. In clinical practice, retreatment is generally performed for more than three weeks after the primary endodontic treatment, potentially resulting in a stronger bond between the root canal dentin and the sealer. During endodontic retreatment, the use of the instrument with the larger apical size is recommended to remove the old filling materials [29,31]. In this study, the root canal was not enlarged using an instrument larger than the one used for the root canal preparation, which was another limitation.

Another limitation was the drying of the root canals with paper points before canal filling. The bond strength of the AH Plus-filled canal increased when the canal was dried, whereas a higher bond strength was obtained in a slightly moist canal with Bio-C Sealer (CSBS) [6]. Further studies are needed to evaluate the retrievability of CSBS under conditions where the setting reaction of the sealer and its interaction with the root canal dentin are maximized.

## 5. Conclusions

The retrievability of filling material during retreatment depends on the specific type of sealer employed during canal filling. The percentages of the removed filling volume were higher in the groups obturated with AHB and CER than in the group obturated with an epoxy resin-based sealer. Both AHB and CER can be retrieved during endodontic retreatment, making them safe options for canal filling. CER appears to be a more suitable bioceramic sealer, as it was observed to form more mineral deposits within dentinal tubules. The supplementary use of an XPF enhanced the removal of the filling material during the retreatment of the oval-shaped canal.

## Figures and Tables

**Table 1:** Root canal sealers used in this study and their composition.

Sealer	Type	Manufacturer	Composition	
			Paste A	Paste B
AH Plus Jet (AHJ)	Epoxy resin-based sealer	Dentsply Sirona, Charlotte, NC, USA	Epoxy resin, calcium tungstate, zirconium oxide, aerosol, iron oxide	1-adamantane amine, N,N’-dibenzyl-5-oxanonandiamin-1,9, tricyclodecane diamine, calcium tungstate, zirconium oxide, aerosol, silicone oil
AH Plus Bioceramic (AHB)	CSBS	Dentsply Sirona, Ballaigues, Switzerland	Zirconium dioxide 50–75%, tricalcium silicate 5–15%, dimethyl sulfoxide 10–30%, lithium carbonate < 0.5%, thickening agent < 6%	
Ceraseal (CER)	CSBS	Metabiomed, Cheongju, South Korea	Zirconium dioxide 45–50%, tricalcium silicate 20–30%, dicalcium silicate 1–10%, tricalcium aluminate 1–10%, thickening agent	

**Table 2:** Initial filling volume, residual filling volumes after retreatment with WOG, and after supplementary use of XPF (mm^3^; mean ± standard deviation).

	Initial Filling Volume			Residual Filling Volume After Retreatment with WOG			Residual Filling Volume After Supplementary Use of XPF		
group	AHJ	AHB	CER	AHJ	AHB	CER	AHJ	AHB	CER
Apical	1.36 ± 0.54	1.19 ± 0.49	1.7 ± 0.53	0.57 ± 0.22	0.44 ± 0.36	0.58 ± 0.3	0.28 ± 0.1 ^b^	0.16 ± 0.1 ^a^	0.19 ± 0.07 ^a,b^
Middle-cervical	9.2 ± 2.73	9.61 ± 2.65	9.57 ± 2.32	1.95 ± 0.9	1.34 ± 0.9	1.45 ± 0.84	1.07 ± 0.52 ^b^	0.39 ± 0.24 ^a^	0.63 ± 0.39 ^a^
Total canal	10.56 ± 2.95	10.79 ± 2.73	11.26 ± 2.47	2.52 ± 0.97	1.78 ± 1.08	2.03 ± 0.99	1.35 ± 0.55 ^b^	0.55 ± 0.21 ^a^	0.82 ± 0.35 ^a^

Different superscript letters in each row indicate significant differences among the three sealer groups (*p* < 0.05).

**Table 3:** Percentages of the removed filling volumes (%) after retreatment with WOG and supplementary use of XPF (mean ± standard deviation), considering the initial filling volume as 100%.

	After Retreatment with WOG			After Retreatment with WOG Followed by XPF		
Group	AHJ	AHB	CER	AHJ	AHB	CER
Apical	55.1 ± 17.6	65.8 ± 15.9	62.9 ± 18.6	78.3 ± 7.5 ^a^	86.3 ± 6.7 ^b^	87.9 ± 5.7 ^b^
Middle-cervical	78.9 ± 7.7 ^a^	86.9 ± 7.1 ^b^	84.6 ± 7.5 ^a,b^	88.1 ± 5.1 ^a^	95.9 ± 2.6 ^b^	93.2 ± 3.9 ^b^
Total canal	76 ± 7.1	84.4 ± 7.2	81.6 ± 7.7	87.1 ± 4.4 ^a^	94.8 ± 2.1 ^b^	92.5 ± 3.1 ^b^

Different superscript letters in each row indicate significant differences among the three sealer groups (*p* < 0.05).

**Table 4:** Three-way ANOVA results of the percentage of removed filling volume.

Source	Sum of Squares	Degree of Freedom	Mean Squares	F	*p*-Value
Sealer	2018.501	2	1009.251	9.649	<0.001
Root region	8370.105	1	8370.105	80.019	<0.001
Instrument	9137.663	1	9137.663	87.357	<0.001
Sealer × root region	64.183	2	32.092	0.307	0.736
Sealer × instrument	26.541	2	13.271	0.127	0.881
Root region × instrument	1760.533	1	1760.533	16.831	<0.001
Sealer × root region × instrument	36.837	2	18.418	0.176	0.839

**Table 5:** Atomic ratio (%) from EDS result of root canal dentin of representative specimen after supplementary with XPF instrumentation.

	AHJ	AHB	CER
C	25.13	25.78	25.24
N	11.66		13.25
O	33.53	39.09	31.59
F	3.75		4.09
Na	1.99	1.34	1.31
Mg	0.94	1	0.52
P	11.72	16.01	11.19
Ca	11.28	16.78	12.81
total	100	100	100

## Data Availability

The data presented in this study are available on request from the corresponding author.
